# The National Institute on Aging Alzheimer's Disease Family Based Study, an enriched resource for the scientific community

**DOI:** 10.1002/alz70860_107516

**Published:** 2025-12-23

**Authors:** Kelley M. Faber, Cory Weinfeld, Abigail Erickson, Pamela Del Rosario, Madison Potocki, Krystal Encarnacion, Amber O'Reilly, Jenverlys Villamizar, Olga Valdez, Jen Gentsch, Alexis Navarro, Victoria Orr, Sarah Payne, Kelly Horner, Elise A Weamer, Katy Figueroa, Karen Elrayes, Ashley LaRoche, Julia Castro, Malia C. Rumbaugh, Amanda Chan, Dolly Reyes‐Dumeyer, The Alzheimer's Disease Family Based Study

**Affiliations:** ^1^ National Centralized Repository for Alzheimer's Disease and Related Dementias (NCRAD), Indianapolis, IN, USA; ^2^ Columbia University, New York, NY, USA; ^3^ National Centralized Repository for Alzheimer's Disease and Related Dementias (NCRAD), Indiana University School of Medicine, indianapolis, IN, USA; ^4^ Columbia University Irving Medical Center, New York, NY, USA; ^5^ Taub Institute for Research on Alzheimer's Disease and the Aging Brain, Columbia University, New York, NY, USA; ^6^ Ronald M. Loeb Center for Alzheimer's Disease, Icahn School of Medicine at Mount Sinai, New York, NY, USA; ^7^ Mayo Clinic, Rochester, MN, USA; ^8^ Washington University in St. Louis, St Louis, MO, USA; ^9^ Department of Psychiatry, Washington University School of Medicine, St. Louis, MO, USA; ^10^ University of Miami Miller School of Medicine, Miami, FL, USA; ^11^ VA Puget Sound, Seattle, WA, USA; ^12^ Indiana University, indianapolis, MO, USA; ^13^ University of Pittsburgh, Pittsburg, PA, USA; ^14^ University of California, Los Angeles, CA, USA; ^15^ Glenn Biggs Institute for Alzheimer's and Neurodegenerative Diseases, University of Texas Health Science Center, San Antonio, TX, USA; ^16^ Glenn Biggs Institute for Alzheimer's and Neurodegenerative Diseases, University of Texas Health San Antonio, San Antonio, TX, USA; ^17^ National Centralized Repository for Alzheimer's Disease and Related Dementias (NCRAD), Indianapolis, IN, USA; ^18^ Department of Medical and Molecular Genetics, Indiana University School of Medicine, Indianapolis, IN, USA; ^19^ Department of Neurology, Vagelos College of Physicians and Surgeons, Columbia University, New York, NY, USA; ^20^ G.H. Sergievsky Center, Columbia University, New York, NY, USA; ^21^ G. H. Sergievsky Center, Vagelos College of Physicians and Surgeons, Columbia University, New York, NY, USA; ^22^ Department of Neurology, Vagelos College of Physicians and Surgeons, Columbia University, and the New York Presbyterian Hospital, New York, NY, USA

## Abstract

**Background:**

The National Institute on Aging Alzheimer's Disease Family Based Study (NIA‐ AD FBS) is a multi‐site, longitudinal study aimed to be a data and bio sample resource for investigators worldwide. Since its inception in 2002 the FBS goal has been to promote greater cooperation and sharing of clinical and biological resources among researchers.

**Methods:**

The focus of the Family Based Study (FBS) has been the recruitment of families with at least two affected individuals and a third first degree relative with or without dementia and willing to participate. Participants are from different ethnic backgrounds including Caucasian, African American and Hispanics. The FBS study is actively recruiting both late onset (LOAD) and early onset Alzheimer's Disease (EOAD) families as well as conducting follow up evaluations every two years, approximately. Uniform assessments are completed across all sites and include DNA, Plasma, PaXgene and PBMC samples collection and brain autopsies whenever possible. We conduct both in person and remote evaluations and use the services of a mobile phlebotomy company to collect bio samples. In addition, we’ve created a protocol for genetic testing on the proband for EOAD families where an additional sample is sent to a CLIA approved lab and after genetic counseling, the presence or not of AD related mutations is disclosed to the families.

**Results:**

To date, this cohort has recruited 1,756 families and acquired data from 9,682 family members. Families are from Caucasian, African Americans and Hispanics ethnic groups. The cohort has longitudinal clinical data, cognitive assessment, family history and bio samples available for sharing. Genotype data includes APOE, GWAS, WES, WGS, brain methylation, RNA sequencing and biomarkers data.

**Conclusion:**

The NIA‐LOAD FBS study is the largest collection of familial Alzheimer's Disease worldwide and many genetic studies of Alzheimer's disease (AD) have included cases and controls from this dataset. Over 140 publications have used data and/or samples from FBS to address the genetics of Alzheimer's Disease. The enriched resources provided by this cohort are invaluable to the scientific community.

